# A cloud-based multi-criteria decision-making framework for green-resilient supplier selection of manufacturing industry

**DOI:** 10.1371/journal.pone.0343165

**Published:** 2026-02-19

**Authors:** Junling Yang, Xiuli Geng, Yuxuan Zhang

**Affiliations:** 1 Chery Commercial Vehicle (Anhui), Co., Ltd., Wuhu, China; 2 Business School, University of Shanghai for Science and Technology, Shanghai, China; Institute of Infrastructure Technology Research and Management, INDIA

## Abstract

The selection of suppliers is crucial for contemporary manufacturing firms to attain their supply chain goals. With the escalation of global environmental concerns, manufacturing companies are facing increasing pressure to assess their suppliers’ environmental performance. To comply with international environmental regulations and align with environmental policies, companies must ensure that their chosen suppliers adhere to green principles. At the same time, given unforeseen circumstances such as natural disasters and political instability, companies increasingly recognize the importance of selecting resilient suppliers to maintain uninterrupted supply chain operations. In contrast, green suppliers are more susceptible to disruptions in the supply chain due to fluctuations in raw material costs and shifts in policy. Accordingly, this paper addresses the issue of selecting green-resilient suppliers. The selection of green-resilient suppliers is a multi-criteria decision-making (MCDM) problem. This study focuses on integrating environmentally sustainable practices and resilient strategies in supplier selection, particularly in the manufacturing industry. First, this paper considers the supplier’s ability to resist risks and environmental protection and constructs a set of evaluation criteria system for selecting suppliers. Second, the C-DEMATEL (Cloud-Decision Making Experimentation and Evaluation Laboratory) method is utilized to ascertain the relative importance of each criterion and to identify the interrelationships among them. Next, the C-TODIM (Cloud-based Portuguese for Interactive Multi-criteria Decision Making) method is proposed to rank suppliers by calculating dominance degree of each potential supplier. Finally, a case study on selecting battery suppliers for a new energy vehicle company is presented to illustrate the proposed approach. Sensitivity analysis and comparative analysis demonstrate the stability and applicability of the proposed framework.

## Introduction

The business management paradigm has quickly shifted in recent years from individual company competition to supply chain competition [1]. Consequently, supply chain management has become increasingly critical for competitiveness in the current business environment. Collaboration with suppliers is vital in reducing production costs and improving operational efficiency in manufacturing companies. Since the cost of raw materials and components could be as high as 70% of the cost of the finished product, in order to maintain their operations, manufacturing companies must establish close cooperation relationships with suppliers [1]. As a result, the selection of suppliers has become a central decision in developing a manufacturing company.

In the International Yearbook of Industrial Statistics 2023, the United Nations Industrial Development Organization (UNIDO) presents a stark finding. In 2020, global manufacturing activities generated 5.9 billion tons (5.9 Gt) of CO_2_ emissions. In order to address this challenge, the European Union adopted the Carbon Boundary Adjustment Mechanism (CBAM) in October 2023. The CBAM is designed to drive reductions in greenhouse gas (GHG) emissions and global energy efficiency improvements. In this context, the manufacturing industry is compelled to confront the environmental challenges inherent in its supply chain. This necessitates the introduction of more rigorous eco-standards and regulations to screen and regulate green suppliers [2]. Therefore, the current supply chain must be transformed into a sustainable operation model, with the identification of eco-conscious suppliers playing a pivotal role in this endeavor.

The inherent characteristics of green suppliers, such as their reliance on specialized materials, stricter regulatory compliance, and often geographically concentrated sustainable sources, make resilience an especially critical factor in assessing the long-term stability and environmental sustainability of supply chains. Unlike conventional suppliers, green suppliers are frequently more vulnerable to disruptions caused by resource scarcity, geopolitical changes, or sudden shifts in environmental policies. Therefore, proactively integrating resilience into supplier selection is not merely a contingency measure—it becomes a fundamental prerequisite for maintaining consistent operations and upholding sustainability commitments in the face of escalating global uncertainties. The concept of resilience has spanned several scientific fields, from psychology to engineering. Resilience has been described as (1) The strength and buffering mechanisms of a system before it is about to change state due to a disorder [3]. (2) The ability of a system to adapt and adjust to a disordered state [4]. (3) the “rebounding” of units to achieve equilibrium after experiencing a disorder [5]. In this paper, the resilience of suppliers is defined as their capacity to rapidly recover from disruptions, which can be understood as the ability to sustain, execute, and restore intended operations despite disruption risks [6].

The process of selecting green-resilient suppliers involves many options, decision-makers, and uncertainty [7]. In general, the selection of suppliers is typically regarded as a multi-criteria decision-making (MCDM) problem, whereby a limited number of suppliers are evaluated based on a predefined set of criteria [8]. Researchers are currently investigating various approaches for evaluating suppliers from a green-resilient standpoint. Amindoust (2018) [9] presented a fuzzy inference system model assurance region data envelopment analysis approach to find potential suppliers’ affinity indices based on sustainability and resilience. Mohammed [10] introduced a quantitative approach aimed at measuring resilience performance metrics, and evaluated the significance of various “resilience” criteria alongside the performance of suppliers in terms of resilience. Mohammed et al. [11] proposed a method for assessing suppliers and determining the ideal order quantity from each one, with a focus on environmentally friendly and resilient attributes. Afrasiabi et al. [12] proposed a combined approach of grey relational analysis and the technique for order preference by similarity to ideal solution (TOPSIS) in a fuzzy context, aimed at evaluating suppliers according to sustainable and resilient standards.

A review of current research has identified several notable research gaps. First, existing studies do not sufficiently account for the variability in how different decision-makers interpret linguistic terms, nor do they adequately address the inherent ambiguity and randomness present in linguistic information processing. The concepts of “green” and “resilience” are inherently complex and difficult to quantify, as they encompass multiple interrelated dimensions and levels of analysis. The use of linguistic expressions to evaluate these concepts may introduce additional uncertainty and imprecision, which can lead to the loss of critical decision-making information. Second, most existing methods for evaluating green-resilient suppliers operate under the assumption of complete rationality—that is, that decision-makers can comprehensively and objectively assess all available information. In practice, however, decision-makers often face constraints such as time limitations, scarce resources, and cognitive biases, causing them to exhibit bounded rationality rather than full objectivity. Third, many existing MCDM methods inadequately consider the influential relationships among supplier evaluation criteria, often neglecting how these interactions affect the overall assessment results.

For the first gap, this paper considers ambiguity and randomness using a cloud model to process the linguistic evaluation information. Two main types of uncertainty associated with natural languages are randomness and ambiguity [13]. There may be differences in understanding linguistic terms among decision-makers in the linguistic decision-making process. The cloud model is designed to describe the ambiguity of qualitative concepts and normal (Gaussian) membership functions while capturing the randomness of the normal distribution. Through its unique three numerical features, it realizes an objective interchangeable transformation between qualitative concepts and quantitative values [14]. In comparison to other models, such as the membership function linguistic model [15] and the 2-tuple linguistic model [16], the cloud model exhibits distinctive advantages in addressing complex and nebulous qualitative concepts. The cloud model considers the vagueness and randomness of linguistic information comprehensively and avoids the loss of decision-making information during information processing. Consequently, it appears more comprehensive and reliable in dealing with complex decision-making problems.

For the second and third gaps, this study aims to propose a novel fuzzy MCDM method based on TODIM (an acronym in Portuguese of Interactive and Multicriteria Decision Making) and DEMATEL (Decision Making Experimentation and Evaluation Laboratory) methods. To begin with, a comprehensive evaluation criteria system of green-resilient supplier consisting of multiple dimensions is established on the basis of literature review. This study adopts the DEMATEL method to capitalize on its distinctive strengths in capturing the interrelationships among evaluation criteria and integrating domain expertise from decision-makers. By structuring complex interactions into an cause-effect model, DEMATEL facilitates a systematic derivation of criterion weights that reflect both expert judgment and the underlying influential networks among factors [17]. Finally, this study utilizes the TODIM method to evaluate potential suppliers, leveraging its capability to incorporate decision-makers’ psychological behaviors and risk preferences without requiring predefined reference points [18,19]. By allowing the explicit modeling of subjective risk attitudes through adjustable attenuation factors, TODIM captures perceived gains and losses under uncertainty from a dynamic reference perspective. This offers a behaviorally realistic framework that adapts to evolving evaluation scenarios and supports robust decision-making without dependence on attribute expectation levels.

We propose a solution approach that combines C-DEMATEL (Cloud-Decision Making Experimentation and Evaluation Laboratory) and C-TODIM (Cloud-an acronym in Portuguese for Interactive Multi-criteria Decision Making) in an effective way. An integrated C-DEMATEL-TODIM framework is proposed in this paper. This novel framework effectively combines cloud model theory with DEMATEL and TODIM to overcome key limitations in existing literature, such as the neglect of cognitive fuzziness and randomness in linguistic evaluations, the oversight of influential relationships among criteria, and the lack of psychological factors in decision-making. The proposed approach begins by characterizing qualitative linguistic information through cloud models, then employs C-DEMATEL to determine criterion weights by capturing causal interrelationships between indicators. Finally, the C-TODIM method is applied to rank suppliers by incorporating decision-makers’ psychological preferences and risk perceptions under uncertainty. This integrated framework offers a more robust and behaviorally realistic decision-support tool for sustainable supply chain management.

The rest of the paper is structured as follows. In the second part, the literature on green-resilient supplier selection is summarized. The third part establishes the green-resilient suppliers’ evaluation attribute system, introduces the basic concept of cloud modeling, and introduces the C-DEMATEL-TODIM method. The fourth part puts the strategy in this paper to use when choosing a battery supplier for the new energy vehicle X firm. The effectiveness of this paper’s method is verified through sensitivity analysis and comparative analysis. The last part summarizes the whole paper.

## Literature review

With increasing environmental awareness among industrial organizations and in the post-pandemic context, a growing number of studies now incorporate both green and resilience criteria as critical factors in supplier selection. Resiliency and green techniques have been explored independently and in certain cases jointly in supplier selection [20,21].

### Green supplier selection

Previous research studies on traditional criteria are more extensive than those on the less established green supplier selection. Büyüközkan and Çifçi [22] used fuzzy DEMATEL, fuzzy ANP, and fuzzy TOPSIS to evaluate green suppliers for a major manufacturing company, namely Ford Otosan. Gupta and Barua [23] addressed the issue of choosing an environmentally friendly supplier for small and medium-sized businesses using a mix of BWM and fuzzy TOPSIS. Khan et al. [24] proposed an MCDM method specifically designed to assess the sustainable performance of suppliers. The Fuzzy-Shannon Entropy method was used to measure the relative significance of sustainability criteria, the introduction of a fuzzy-inference system to assess and prioritize suppliers. Babbar and Amin [25] introduced a framework for order quantity allocation and supplier selection, based on a stochastic multi-objective mathematical model and a two-step fuzzy quality function deployment (QFD) method. Musa et al. [26] combined cubic Pythagorean fuzzy contexts with TOPSIS to select green suppliers. Wu et al. [27] provided an integrated methodology to address green supplier selection problems based on the BWM and the VIKOR technique in an interval type-2 fuzzy environment. Jain and Singh [28] used fuzzy interference systems (FIS) and the fuzzy kano philosophy of sustainable environments to provide a two-phase selection strategy for choosing sustainable suppliers for large-scale companies. Khattak et al. [29] incorporated management opinion into a green supplier selection model using quality function deployment and interactive fuzzy programming. Chen et al. [30] proposed a novel framework to identify smart-sustainable SCMP (supply chain management practices) as supplier selection criteria for an intelligent supply chain. It also proposed a hybrid rough-fuzzy DEMATEL-TOPSIS approach to sustainable supplier selection for an intelligent supply chain. Stevic et al. [31] presented a MCDM model called Measurement of Alternatives and Ranking according to Compromise Solution (MARCOS) for addressing sustainable supplier selection issues in the private medical industry. Mahmoudi et al. [32] conducted research on the process of choosing a sustainable supplier for a major capital project, taking into consideration several rankings of criteria and options by using the ordinal priority approach and grey systems theory. Gai et al. [33] introduced a two-step approach to address the issue of distributing orders and choosing green suppliers. They used a MCDM model and developed a bi-objective mathematical model to handle the uncertainty inherent in a fuzzy environment.

### Resilient supplier selection

As evidenced above, numerous scholars have investigated the selection of green suppliers. Nowadays, due to the frequent occurrence of emergencies, more and more scholars are focusing on their resilience capability when selecting suppliers. Torabi et al. [34] proposed a fuzzy stochastic bi-objective optimization model to solve an SS/OA problem and improve supply chain resilience under operational and disruption risks. Rajesh and Ravi [35] used grey relational analysis and linguistic assessment to prioritize supplier selection in a resilient supply chain context. Sahu et al. [36] utilized a decision-making procedural hierarchy and VIKOR method integrated with fuzzy set theory to evaluate and select resilient suppliers in supply chains. Pramanik et al. [37] utilized a fuzzy-multi-criterion group decision-making approach, integrating TOPSIS, AHP, and QFD methodologies, to develop a resilient supplier selection process in a manufacturing system. Zhang et al. [38] applied a fuzzy DEMATEL–ISM method to identify and structure the key factors influencing resilient supplier selection in agri-food supply chains. Parkouhi and Ghadikolaei [39] provided a resilience-based conceptual model for supplier selection, defined essential characteristics of robust supplier selection and supplier resilience levels through expert opinion, and utilized a fuzzy MCDM model to find the most resilient suppliers. Davoudabadi et al. [40] developed a resilient supplier selection method that converts the decision maker’s linguistic variable judgments into interval-valued intuitionistic fuzzy numbers, determines criterion weights using entropy indices, and ranks suppliers through a complex proportionality assessment methodology. Parkouhi et al. [41] proposed a model combining grey theory, DEMATEL, and SAW methods to select resilient suppliers in uncertain conditions. Davoudabadi et al. [42] presented a new integrated efficiency measurement model combining statistical techniques, decision-making, and mathematical programming for resilient supplier analysis. The research mentioned above has led academics to concentrate on supplier resilience in supplier selection due to the growing occurrence of catastrophes. This allows organizations to enhance their supply chain resilience while dealing with interruptions and emergencies.

### Green-resilient supplier selection

However, considering resilience or green alone can no longer meet the needs of manufacturing companies, some studies combine them. Sen et al. [43] introduced a unique concept of performance index known as the “g-resilience” index to help evaluate the performance of suppliers in order to select the best candidate suppliers. Sen et al. [44] proposed a decision support framework using fuzzy set theory for green and resiliency-based supplier selection, introducing a simplified dominance-based approach and a g-resilient index for performance assessment. Fallahpour et al. [45] developed a hyper-hybrid fuzzy supplier performance decision-making framework based on resilient-sustainable criteria tailored explicitly for the Malaysian palm oil industry, contributing to the understanding and integrating resilience and sustainability concepts in supplier selection processes. Alimohammadlou and Khoshsepehr [46] used hesitant fuzzy analysis to rate G-resilient providers on different characteristics. This provides insights for organizations on selecting suppliers with reduced environmental impact and enhanced performance. Amindoust [9] proposed an approach using a developed modular FIS model to determine the affinity indices of candidate suppliers based on their resiliency and sustainability, followed by applying AR-DEA to rank the given set of suppliers. Mohammed [10] proposed a quantitative methodology to quantify resilience performance metrics by assessing the relative importance of “resilience” criteria and supplier resilience performance through the MCDM methodology. Mohammed et al. [11] presented a method for assessing suppliers and determining the most suitable order amount from each supplier based on their green and resilient attributes. Afrasiabi et al. [12] proposed a complete framework that incorporates robust, well-defined fuzzy MCDM techniques for evaluating suppliers according to resilient and sustainable standards. Saghafinia et al. [47] applied the fuzzy DEA method to select green-resilient suppliers in the home appliance industry. Zhang et al. [48] applied the fuzzy DEA method to select green-resilient suppliers. Abedian et al. [49] applied the fuzzy DEA method to select green-resilient suppliers in electronic manufacturing systems. Table 1 offers a concise summary of the studies mentioned above, highlighting their salient features and the hybrid methodologies employed.

**Table 1:** Summary of relevant studies on green and resilient supplier selection methodologies.

Researchers	Methodology	Advantage				
		Randomness	Ambiguity	Interaction between criteria	The psychological behavior of decision-makers	Reduce computational difficulty
Sen et al. (2016)	FUZZY-TODIM		✓		✓	
Sen et al. (2017)	Fuzzy set theory + simplified TODIM and PROMETHEE	✓	✓		✓	✓
Fallahpour et al. (2021)	FDEMATEL + FBWM + FANP + FIS		✓	✓		
Alimohammadlou et al. (2022)	Hesitant Fuzzy BWM		✓	✓		
Amindoust (2018)	AR-DEA		✓			✓
Mohammed (2020)	DEMATEL-VIKOR			✓		
Mohammed (2021)	AHP-TOPSIS					
Saghafinia et al. (2024)	FUZZY-DEA		✓			✓
Zhang et al. (2024)	Z-BWM-TOPSIS		✓			✓
Abedian M et al. (2023)	FUZZY-DEA					
Afrasiabi et al. (2022)	FBWM + fuzzy GRA-TOPSIS		✓	✓	✓	✓

Table notes: A comparative analysis of decision-making methods shows how different approaches handle uncertainty, ambiguity, criteria interactions, The psychological behavior of decision-makers, and computational complexity.

In summary, numerous studies have explored supplier selection from green and resilient perspectives, emphasizing their significance and providing a foundation for our current research. However, there are still some research gaps in existing studies: decision-makers have different understandings of linguistic terminology and fail to consider the ambiguity and randomness of linguistic information. The evaluation methods ignore the psychological behavior of decision-makers under conditions of limited rationality. Furthermore, the inherent complexity of some MCDM methods has discouraged managers from adopting them, reducing both willingness and efficiency. Therefore, this paper proposes a C-DEMATEL-TODIM framework that addresses the randomness and ambiguity in decision-making information, accounts for interactions among criteria and the psychological behavior of decision-makers, while simultaneously reducing computational complexity.

## Proposed decision-making framework

**Fig 1:**
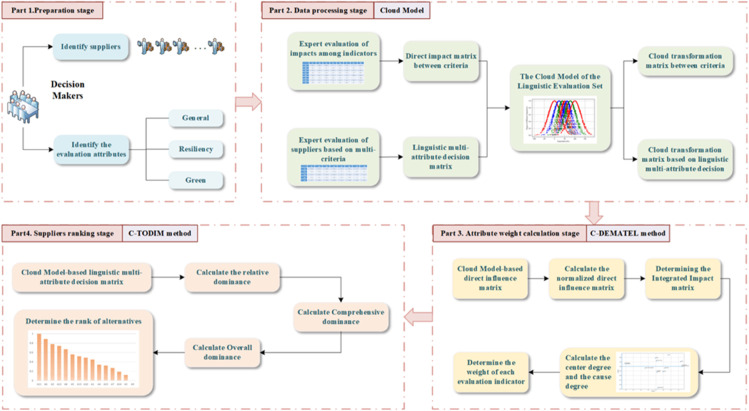
The framework for green-resilient supplier selection.

To conduct a scientifically-grounded evaluation of suppliers that integrate both green and resilient attributes, a structured decision-making framework is proposed, as illustrated in Fig 1. The framework consists of four main components: The first part addresses the selection and definition of green-resilient criteria. The second part provides the foundational concepts of cloud model. The third and fourth parts present the C-DEMATEL and C-TODIM methods, respectively, which together form the integrated C-DEMATEL-TODIM approach proposed in this study.

### Identification of green-resilient criteria for supplier selection

The green-resilient criteria encompass a wide range of quantitative and qualitative factors. To effectively choose the most suitable supplier in the manufacturing field, it is crucial to carefully select an evaluation criterion that is well-suited to the practical problems at hand. Three dimensions for evaluating suppliers and their sub-criteria were identified in domestic and international studies on supplier evaluation and combining expert opinions. Details are shown in Table 2.

**Table 2:** Evaluation criteria for green-resilient suppliers.

Criteria	Sub-criteria	Definition	Authors
General	Quality (C1)	This feature shows that the purchased components meet the automotive manufacturer’s quality standards, as demonstrated through quality certifications and compliance with international quality management system standards.	(Amindoust, 2018) [9]
	Cost (C2)	This feature shows that the supplier offers reasonably priced components, allowing for optimal cost-effectiveness through price negotiations.	(Hosseini and Al Khaled, 2019) [1]
	Delivery (C3)	This feature shows that the supplier ensures timely delivery of components, enabling smooth operation of the production line and avoiding capacity loss and delays.	(Amindoust, 2018) [9]
	Service (C4)	This feature shows that the supplier provides excellent after-sales service, promptly addressing and resolving quality issues or technical support needs.	(Hoseini et al., 2022) [50]
Resiliency	Robustness (C5)	This feature shows that the chosen components are durable and reliable, ensuring the long-term reliability and safety of the vehicles.	(Fallahpour et al., 2021) [45]
	Responsiveness (C6)	This feature shows that effective communication channels are established with the supplier, enabling quick responses to urgent requirements or change requests.	(Nazari-Shirkouhi et al., 2023) [51]
	Cooperation (C7)	This feature shows that cooperative relationships are fostered with suppliers, promoting information sharing, technological collaboration, and joint problem-solving to enhance supply chain efficiency and quality.	(Hasan et al., 2020) [52]
	Restorative capacity (C8)	This feature shows that the supplier can repair and maintain components, minimizing failures and downtime.	(Fallahpour et al., 2021) [45]
Green	Green design capability (C9)	This feature shows that priority is given to suppliers with green design capabilities, reducing adverse environmental impacts through the use of recyclable materials or energy-efficient designs.	(Sen et al., 2017) [44]
	Green Image (C10)	This feature shows that suppliers with a robust environmental image are selected, enhancing the automotive brand’s environmental reputation and market competitiveness.	(Fallahpour et al., 2021) [45]
	Pollution control (C11)	This feature shows that automotive component suppliers comply with industry-specific environmental regulations and implement effective pollution control measures, reducing emissions and waste generation.	(Afrasiabi et al., 2022) [12]
	Environmental management system (C12)	This feature shows that suppliers establish and implement environmental management systems tailored to the automotive components industry, including monitoring and improving environmental performance and compliance with industry-specific environmental standards.	(Kayani et al., 2023) [53]

Table notes: This table provides definitions and corresponding authors for various criteria and sub-criteria used in supplier selection processes.

### Background information of the cloud model

When converting qualitative information into uncertain data, the cloud model can not only take into account the ambiguity and randomness of qualitative information based on the three numerical characteristics of expectation (Ex), entropy (En), and hyper entropy (He). Moreover, by leveraging a normal distribution-based algorithm, the model minimizes information loss during conversion, thereby supporting more reliable and information-preserving decision-making [54]. Some relevant definitions of cloud model are given below.

**Definition 1.** [55] Let *U* be the universe of discourse, and *T* be a qualitative concept. If a random instantiation of the qualitative concept *T* is the element *x* (x∈X), which satisfies $x\sim 𝒩(Ex,En^{\prime 2}),\;En^{\prime }\sim 𝒩(En,He^{2}),$ and y∈[0,1] is the certainty degree of *x* belonging to the qualitative concept *T*, which satisfies

$$y=e^{-\frac{(x-Ex{)}^{2}}{2(En^{\prime }{)}^{2}}}.$$ (1)

The distribution of variable *x* in the universe *U* is referred to as a *normal cloud*, and the occurrence of a data point within this cloud can be represented as (*x*,*y*).

Three numerical features can accurately represent all the quantitative attributes of a concept within a cloud. Examples include the elements *Ex*, *En*, and *He*. The symbol “*Ex*” represents the mathematical expectation that the positions of cloud droplets correspond to a concept within the universe. It can be seen as the most outstanding and typical example of the qualitative concept. *En* is a metric that quantifies the level of uncertainty associated with a qualitative concept, specifically involving randomness and ambiguity. *He* measures the degree of uncertainty in *En*, specifically, the second-order entropy of the entropy. Cloud *K* can be represented as K=(Ex,En,He), where *Ex*, *En*, and *He* are three numerical characteristics.

**Fig 2:**
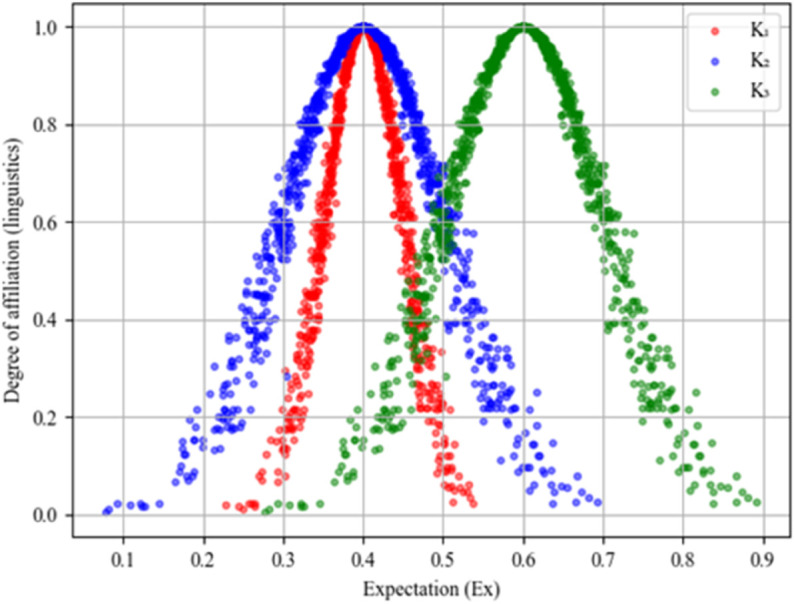
Comparison diagram of cloud model.

A comparison diagram of cloud models $K_{1}=(0.4,\;0.05,\;0.005)$, $K_{2}=(0.4,\;0.1,\;0.01)$, and $K_{3}=(0.6,\;0.1,\;0.01)$ for the number of cloud droplets *n* = 1000 is shown in Fig 2. *K*_1_ has the same *Ex* as *K*_2_, and *K*_2_ has a larger *En* and *He*, which are larger in the diagram in terms of their span and thickness. *K*_2_ has the same *En* and *He* as *K*_3_, and *K*_3_ has a larger *Ex*, indicating that its qualitative conceptual expectations are larger and are positioned further right in the diagram.

**Definition 2.** [56] Let $H=\{h_{i}|i=-n,\ldots ,0,\ldots ,n\}$ (n∈ℕ) be a finite linguistic term set with odd cardinality, where *h*_*i*_ represents a possible value of a linguistic variable. Then, define the exact number of scores for *h*_*i*_ as $\theta_{i}$ ($\theta_{i}\in [0,1]$) with the computational formula:

$$\theta_{i}=\left\{ \begin{array}{cc} \frac{a^{n}-a^{-i}}{2a^{n}-2}, & -n\leq i\leq 0\\ \frac{a^{n}+a^{i}-2}{2a^{n}-2}, & 0\leq i\leq n \end{array}\right.$$ (2)

The value of *a* can be obtained experimentally or subjectively. When the linguistic term set has cardinality 2n+1=7, typically a≈1.37. When the linguistic term set has cardinality 2*n* + 1 = 9, typically a≈1.40.

**Definition 3.** [57] Let $U=[X_{\min},X_{\max}]$ be the universe of discourse, the cloud model after the transformation of linguistic variable *h*_*i*_ is defined as $M_{i}=K_{i}(Ex_{i},En_{i},He_{i})$

and the computation process is as follows:

**Step 1.** Calculate $\theta_{i}$ according to Eq (2).

**Step 2.** Calculate *Ex*_*i*_ by the following equation:

$$Ex_{i}=X_{\min}+\theta_{i}\left(X_{\max}-X_{\min}\right).$$ (3)

**Step 3.** Calculate *En*_*i*_ by the following equations:

$$En_{i}^{\prime }=\left\{ \begin{array}{cc} \frac{\left(1-\theta_{i})(X_{\max}-X_{\min}\right)}{3}, & -n\leq i\leq 0,\\ \frac{\theta_{i}(X_{\max}-X_{\min})}{3}, & 0\leq i\leq n, \end{array}\right.$$ (4)

$$En_{i}=En_{-i}=\left\{ \begin{array}{cc} \frac{\left(\theta_{i}+2\theta_{i+1})(X_{\max}-X_{\min}\right)}{9}, & i=0,\\ \frac{\left(\theta_{i-1}+\theta_{i}+\theta_{i+1})(X_{\max}-X_{\min}\right)}{9}, & |i|\leq n-1,\\ \frac{\left(\theta_{i-1}+\theta_{i})(X_{\max}-X_{\min}\right)}{6}, & |i|=n, \end{array}\right.$$ (5)

**Step 4.** Calculate *He*_*i*_ by the following equation:

$$He_{i}=He_{-i}=\frac{\max_{k}\left\{En_{k}^{\prime }-En_{i}\right\}}{3}.$$ (6)

**Definition 4.** [57] Let $M_{1}=(Ex_{1},En_{1},He_{1})$ and $M_{2}=(Ex_{2},En_{2},He_{2})$ be any two clouds in the same domain. Then, the operational rules for *M*_1_ and *M*_2_ are defined as follows:

$$M_{1}+M_{2}=(Ex_{1}+Ex_{2},\;\sqrt{En_{1}^{2}+En_{2}^{2}},\;\sqrt{He_{1}^{2}+He_{2}^{2}}).$$ (7)

$$M_{1}\times M_{2}=(Ex_{1}Ex_{2},\;\sqrt{(En_{1}Ex_{2}{)}^{2}+(En_{2}Ex_{1}{)}^{2}},\;\;\;\sqrt{(He_{1}Ex_{2}{)}^{2}+(He_{2}Ex_{1}{)}^{2}}).$$ (8)

$$\lambda M_{1}=(\lambda Ex_{1},\;\sqrt{\lambda }\;En_{1},\;\sqrt{\lambda }\;He_{1}).$$ (9)

$$M_{1}^{\lambda }=(Ex_{1}^{\lambda },\;\sqrt{\lambda }\;Ex_{1}^{\lambda -1}En_{1},\;\sqrt{\lambda }\;Ex_{1}^{\lambda -1}He_{1}).$$ (10)

**Definition 5.** [58] For a set of cloud droplets (*x*,*y*), the score *s* = *xy* is defined as the *first-order scoring* of the cloud droplet with respect to the qualitative concept *T*. The mathematical expectation $\hat{s}$ (or the median $s_{\text{mid}}$ of the score) is defined as the *total score* of the cloud with respect to the qualitative concept *T*.

Based on the aforementioned calculation method of the total score for clouds, it is possible to compare the sizes of any two clouds $M_{1}=(Ex_{1},En_{1},He_{1}),\;M_{2}=(Ex_{2},En_{2},He_{2})$ within the same domain. Let ${\hat{s}}_{1}$ and ${\hat{s}}_{2}$ denote their respective total scores. Then:

- If ${\hat{s}}_{1}\geq {\hat{s}}_{2}$, it implies that *M*_1_ is greater than or equal to *M*_2_ ($M_{1}\geq M_{2}$);
- If ${\hat{s}}_{1}<{\hat{s}}_{2}$, it implies that *M*_1_ is less than *M*_2_ ($M_{1}<M_{2}$).

Furthermore, the *cloud distance* is defined to measure the distance between clouds.

**Definition 6.** [58] Let $M_{1}=(Ex_{1},En_{1},He_{1})$ and $M_{2}=(Ex_{2},En_{2},He_{2})$ be any two clouds in the same domain. The *Hamming distance* between *M*_1_ and *M*_2_ is defined as follows:

$$d(M_{1},M_{2})=|\left(1-\frac{En_{1}^{2}+He_{1}^{2}}{En_{1}^{2}+He_{1}^{2}+En_{2}^{2}+He_{2}^{2}}\right)Ex_{1}\;\;-\left(1-\frac{En_{2}^{2}+He_{2}^{2}}{En_{1}^{2}+He_{1}^{2}+En_{2}^{2}+He_{2}^{2}}\right)Ex_{2}|,\;d(M_{1},M_{2})\in [0,+\infty ].$$ (11)

**Definition 7.** Let $M_{1}=(Ex_{1},En_{1},He_{1})$ and $M_{2}=(Ex_{2},En_{2},He_{2})$ be any two clouds in the same domain. Then the *normalized distance* between *M*_1_ and *M*_2_ is defined as:

$$d^{\prime }(M_{1},M_{2})=\frac{d(M_{1},M_{2})}{d(\max M,\min M)},\;d^{\prime }(M_{1},M_{2})\in [0,1].$$ (12)

Here, d(maxM,minM) denotes the Hamming distance between the largest and smallest clouds in the qualitative concept:

$$d(\max M,\min M)=|\left(1-\frac{En_{+t}^{2}+He_{+t}^{2}}{En_{+t}^{2}+He_{+t}^{2}+En_{-t}^{2}+He_{-t}^{2}}\right)En_{+t}\;\;-\left(1-\frac{En_{\frac{-(n-1)}{2}}^{2}+He_{\frac{-(n-1)}{2}}^{2}}{En_{+t}^{2}+He_{+t}^{2}+En_{-t}^{2}+He_{-t}^{2}}\right)En_{-t}|.$$ (13)

Since $En_{+t}=En_{-t}$ and $He_{+t}=He_{-t}$, the above equation can be simplified as:

$$d(\max M,\min M)=\frac{En_{+t}-En_{-t}}{2}=\frac{X_{\max}-X_{\min}}{2}.$$ (14)

In summary, the normalized distance between *M*_1_ and *M*_2_ can be simplified as:

$$d^{\prime }(M_{1},M_{2})=\frac{2d(M_{1},M_{2})}{X_{\max}-X_{\min}}.$$ (15)

It should be noted that this paper normalizes the existing cloud Hamming distances, so that the normalized cloud distances can satisfy the basic characteristics of distances and provide a cloud similarity determination method that is easy to calculate. Therefore, the cloud-normalized distance given in this paper is more reasonable.

### Calculation of criteria weights based on C-DEMATEL

When determining criteria weights, the traditional DEMATEL method uses exact numbers to characterize and portray the interactions between criteria. However, decision-makers usually use linguistic evaluation sets when assessing the interactions between criteria, and the variability between neighboring linguistic phrases should be different. Therefore, this paper uses a cloud model to characterize and portray the DEMATEL scores given by decision-makers on the basis of previous research. The following steps enhance the criteria weighting process based on the C-DEMATEL method.

1. **Construct the cloud model-based direct influence matrix *P*.** The cloud model-based direct influence matrix *P* is built by transforming the decision-makers’ language phrase scores into cloud model scores, as outlined in Definition 3:
  $$P=(p_{gh}{)}_{m\times m}=(\begin{array}{cccc} p_{11} & p_{12} & \cdots & p_{1m}\\ p_{21} & p_{22} & \cdots & p_{2m}\\ \vdots & \vdots & \ddots & \vdots \\ p_{m1} & p_{m2} & \cdots & p_{mm} \end{array})$$ (16)
  Here, the cloud model score *p*_*gh*_ assigned by the decision-maker represents the impact of the *g*th criterion on the *h*th criterion.
2. **Calculate the normalized direct influence matrix *Z*.** A cloud model scoring function based on the ideal point method is provided to transform the cloud model score *p*_*gh*_ into a precise numerical score *y*_*gh*_. Based on this, the normalized direct influence matrix $𝐙=(z_{gh}{)}_{m\times m}$ is calculated as:
  $$Z=(z_{gh}{)}_{m\times m}=\{\begin{array}{l} p_{gh}=\frac{d\left(p_{gh},\min M\right)}{d\left(p_{gh},\min M\right)+d\left(p_{gh},\min M\right)}\\ z_{gh}=p_{gh}/\max_{\begin{array}{c} g=1,2,\cdots ,m \end{array}}\left(\sum_{h=1}^{m}p_{gh}\right) \end{array}$$ (17)
  Here, $d(p_{gh},\max M)$ is the Hamming distance between *p*_*gh*_ and the largest cloud in the qualitative concept, and $d(p_{gh},\min M)$ is the Hamming distance between *p*_*gh*_ and the smallest cloud in the qualitative concept.
3. **Determine the integrated impact matrix *T*.** Following the traditional method, the integrated impact matrix $𝐓=(t_{gh}{)}_{m\times m}$ is calculated as:
  $$𝐓=\lim_{r\to \infty }\left({𝐙}^{1}+{𝐙}^{2}+\cdots +{𝐙}^{r}\right)\;=𝐙{\left(𝐈-𝐙\right)}^{-1},$$ (18)
  where **I** is the unit matrix whose diagonal elements are 1 and the rest are 0.
4. **Calculate the center degree and the cause degree.** First, calculate the sum of the elements of each row and each column of the integrated impact matrix **T**:
  $$e_{g}=\sum_{h=1}^{m}t_{gh},\;f_{g}=\sum_{g=1}^{m}t_{gh}.$$ (19)
  Let *g* = *h* = *j*. Then, the *centrality* of the *j*th criterion is $\left(e_{j}+f_{j}\right)$, characterizing the relative importance of the criterion in the system; and the *causality* is $\left(e_{j}-f_{j}\right)$, characterizing the strength of its causal relationship with other criteria.
5. **Determine the weight of each evaluation criterion *w***_***j***_. The calculation formula is:
  $$w_{j}=\frac{\sqrt{(e_{j}+f_{j}{)}^{2}+(e_{j}-f_{j}{)}^{2}}}{\sum_{j=1}^{m}\sqrt{(e_{j}+f_{j}{)}^{2}+(e_{j}-f_{j}{)}^{2}}},$$ (20)
  where *w*_*j*_ is the weight of the *j*th evaluation criterion.

### Evaluation of suppliers based on C-TODIM

**For the linguistic multi-criteria decision-making problem,** assume that the set of criteria is


$$C=\left(C_{1},C_{2},\cdots ,C_{m}\right),$$

where $C_{j}\;(j=1,2,\cdots ,m)$ is the *j*th criteria; the set of suppliers is


$$𝔸=\left(A_{1},A_{2},\cdots ,A_{n}\right),$$

where $A_{i}\;(i=1,2,\cdots ,n)$ is the *i*th supplier; and the multi-criteria decision-making matrix is


$$𝕏={\left(x_{ij}\right)}_{m\times m},$$

where *x*_*ij*_ is the linguistic evaluation information given by the decision-maker for supplier *A*_*i*_ with respect to criteria *C*_*j*_. For the integrated weighted cloud decision matrix $𝕏={\left(x_{ij}\right)}_{m\times m}$ and the criteria weights *w*_*j*_ obtained based on C-DEMATEL, the normalized cloud distance is introduced into the process of calculating the dominance degree of the supplier. The C-TODIM is proposed to rank suppliers by dominance. The specific steps are as follows:

1. **Calculation of relative weights of criteria**
  Let w^*^ be the selected reference criteria and the relative weight of criteria *w*_*j*_ be denoted as $w_{j}^{\prime }$:
  $$w_{j}^{\prime }=\frac{w_{j}}{w^{*}},\;j=1,2,\cdots ,m$$ (21)
  where $w^{*}=\max\{w_{1},w_{2},\cdots ,w_{m}\}$. C^*^ represents the criteria corresponding to w^*^.
2. **Calculate the degree of dominance $\phi_{il}^{j}=\phi_{j}(A_{i},A_{l})$ of supplier *A***_***i***_ **over supplier *A***_***l***_ **under criteria *C***_***j***_:
  $$\phi_{j}(A_{i},A_{l})=\left\{ \begin{array}{cc} w_{j}^{\prime }\cdot d(x_{ij},x_{lj}), & x_{ij}>x_{lj}\\ 0, & x_{ij}=x_{lj}\\ -\frac{1}{\theta }w_{j}^{\prime }\cdot d(x_{ij},x_{lj}), & x_{ij}<x_{lj} \end{array}\right.$$ (22)
  where $d(x_{ij},x_{lj})$ refers to the normalized cloud distance as defined in Definition 7. *θ* is the loss recession coefficient and θ>0. If 0<θ<1, the effect of loss increases; if θ>1, the effect of loss decreases [59].
3. **Create a matrix of dominance degrees under each decision criteria:**
  $$\phi_{il}^{j}=0,\;i=1,2,\cdots ,n,\;l=1,2,\cdots ,n,\;j=1,2,\cdots ,m$$ (23)
4. **Calculate the degree of dominance of supplier *A***_***i***_ **over supplier *A***_***l***_:
  $$\delta (A_{i},A_{l})=\sum_{j=1}^{m}\phi_{il}^{j}$$ (24)
5. **Calculate the overall dominance degree *T*(*A***_***i***_**) of supplier *A***_***i***_ **and rank the suppliers:**
  $$T(A_{i})=\frac{\sum_{l=1}^{n}\delta (A_{i},A_{l})-\min_{1\leq i\leq m}\sum_{l=1}^{n}\delta (A_{i},A_{l})}{\max_{1\leq i\leq m}\sum_{l=1}^{n}\delta (A_{i},A_{l})-\min_{1\leq i\leq m}\sum_{l=1}^{n}\delta (A_{i},A_{l})}$$ (25)
  Suppliers are ranked based on the magnitude of *T*(*A*_*i*_), with a higher *T*(*A*_*i*_) indicating a better supplier.

**Ethics statement.** The submission does not require an ethics statement.

## Case study

New energy vehicle company X needs to procure many batteries during the production process. As the core component of new energy vehicles, batteries account for a significant proportion of manufacturing cost and directly affect vehicle performance, driving range, and safety. Consequently, any disruption in the battery supply would immediately cause production delays and threaten the stability of the entire supply chain. Battery supply, however, is vulnerable to multiple sources of risk, including shortages of critical raw materials such as lithium and cobalt, volatility in international trade policies, and unexpected events such as transportation interruptions or supplier shutdowns.

Therefore, when selecting suppliers, the company should consider resilience, which refers to a supplier’s ability to maintain continuous delivery under uncertainty and to recover rapidly from disruptions. In practice, resilience can be reflected in delivery reliability, recovery time, capacity flexibility, and risk-mitigation practices. Taking resilience into account ensures not only the reliability of the supply chain, but also its flexibility, thereby supporting stable and sustainable vehicle production. In addition, compliance with environmental regulations and standards is an indispensable requirement in the battery supply process. Selecting suppliers that adhere to green standards not only fulfills corporate environmental responsibility but also ensures product compliance with local environmental laws.

In summary, when choosing a battery supplier, company X should give priority to two key aspects: resilience and green standards. The former guarantees supply chain reliability and flexibility, while the latter demonstrates corporate commitment to sustainability. Such a dual consideration will help the company build a competitive advantage in the rapidly growing new energy vehicle market. As a research background, this study concentrates on applying the proposed method to rank and select the optimal battery suppliers. A more detailed process of selecting alternative suppliers is presented in the following. The data used in this process can be found in S1 dataset.

### Calculation process and results

Company X intends to select four suppliers (*A*_1_, *A*_2_, *A*_3_, *A*_4_) of electric vehicle batteries. Each of them occupies an important position in China’s new energy vehicle battery market, and each of them has unique technical advantages, market positioning, and development potential. After joint discussions among several experts in supply chain management, a set of criteria, including Quality (*C*_1_), Cost (*C*_2_), Delivery (*C*_3_), Service (*C*_4_), Robustness (*C*_5_), Responsiveness (*C*_6_), Cooperation (*C*_7_), Restorative capacity (*C*_8_), Green design capability (*C*_9_), Green Image (*C*_10_), Pollution control (*C*_11_), and Environmental management system (*C*_12_), were identified for multiple criteria decision-making. Due to the complexity, uncertainty, and fuzziness of objective factors and the subjective nature of human thinking, the expert group chose linguistic evaluation information for decision-making. The linguistic evaluation set is represented by Very Poor (VP), Poor (P), Medium Poor (MP), Fair (F), Medium Good (MG), Good (G) and Very Good (VG), and the evaluation information for the suppliers’ performance is shown in Table 3. Additionally, in the DEMATEL analysis, the influence of each criterion on others is assessed using a seven-point scale: Very Low (VL), Low (L), Medium Low (ML), Medium/Moderate (M), Medium High (MH), High (H), and Very High (VH). Specifically: VL (Very Low) indicates that the effect of one criterion on another is negligible or almost nonexistent; L (Low) indicates a minor influence; ML (Medium Low) represents a somewhat noticeable influence; M (Medium/Moderate) reflects a moderate effect; MH (Medium High) denotes a significant influence; H (High) indicates a strong effect; VH (Very High) represents an extremely strong influence. The specific scale representation of DEMATEL is shown in Table 4. Each level is determined based on expert judgment and literature review, ensuring that the cause-and-effect relationships among criteria are clearly defined and transparent. The evaluation information for the direct influence relationships between the criteria is presented in Table 5. In the following, we will use the proposed method in this paper to select the optimal supplier of electric vehicle batteries.

**Table 3:** The linguistic multi-attribute decision matrix given by the expert group.

Supplier	$C_{1}$	$C_{2}$	$C_{3}$	$C_{4}$	$C_{5}$	$C_{6}$	$C_{7}$	$C_{8}$	$C_{9}$	$C_{10}$	$C_{11}$	$C_{12}$
*A*_1_	VG	VP	VG	G	F	G	F	VP	M	G	VG	P
*A*_2_	VG	MG	G	MG	G	G	VP	G	P	F	VG	VG
*A*_3_	VG	G	MP	VG	MP	VG	P	G	MP	G	VG	G
*A*_4_	G	VG	VP	MP	G	VG	VG	G	MP	G	G	VP

**Table 4:** The scale corresponding to the influence of high and low between attributes in DEMATEL.

Specific description	Very Low	Low	Medium Low	Medium	Medium High	High	Very High
**Scale**	VL	L	ML	M	MH	H	VH

**Table 5:** Direct impact matrix between criteria given by the expert group.

Affecting Criterion	$C_{1}$	$C_{2}$	$C_{3}$	$C_{4}$	$C_{5}$	$C_{6}$	$C_{7}$	$C_{8}$	$C_{9}$	$C_{10}$	$C_{11}$	$C_{12}$
$C_{1}$	VL	VH	VH	L	H	H	L	H	VH	L	MH	H
$C_{2}$	VH	VL	M	VH	VH	VH	VL	H	M	M	VH	VH
$C_{3}$	H	VH	VL	VH	VH	VH	H	VH	VL	VL	VL	H
$C_{4}$	VH	H	VH	VL	VH	VH	M	VH	VH	H	M	VH
$C_{5}$	VH	H	VH	H	VL	H	ML	VH	VL	VL	MH	MH
$C_{6}$	VL	VL	MH	VH	H	VL	L	MH	VL	VL	MH	VH
$C_{7}$	VL	L	M	H	VL	H	VL	VL	H	L	VL	H
$C_{8}$	ML	H	VH	M	VH	MH	VL	VL	L	VL	L	MH
$C_{9}$	L	M	VL	VH	H	VH	L	L	VL	L	L	VH
$C_{10}$	VL	VL	VL	MH	M	VL	VL	VL	VH	VL	VH	H
$C_{11}$	ML	H	M	H	VL	H	VL	H	VH	M	VL	VH
$C_{12}$	MH	VH	H	VH	H	VH	M	H	VH	H	VH	VL

The following is the computational process of the linguistic multi-criteria decision-making method based on C-DEMATEL-TODIM:

**Phase 1: Computing criteria weights using C-DEMATEL**

**Fig 3:**
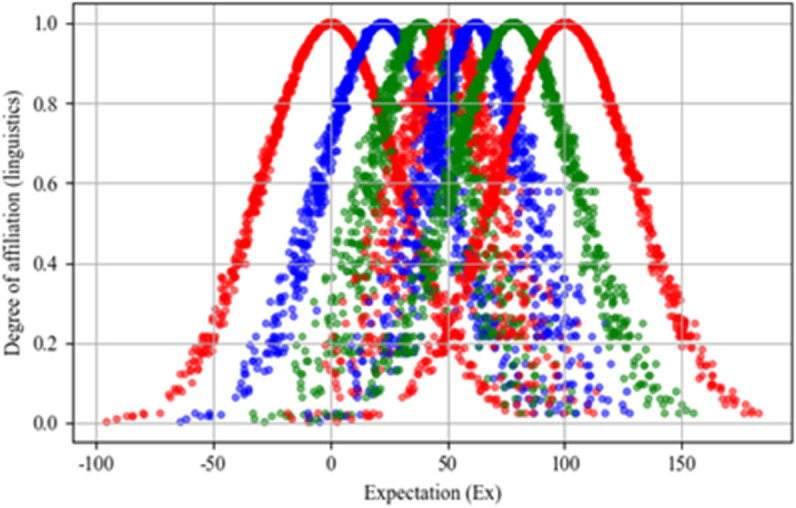
Seven clouds of the linguistic evaluation set.

Based on Eq (3)–(6), using the cloud model representation in Definition 3, when the linguistic term set is $H=\left\{h_{-3},h_{-2},h_{-1},h_{0},h_{1},h_{2},h_{3}\right\}$, then the granularity of the linguistic evaluation set at this point is 7. Assuming that the universe of discourse is U=[0,100], then according to the background information of the cloud model, we can calculate 7 clouds as *C*_−3_(0,29.65,1.228), *C*_−2_(22.1,26.63,2.234), *C*_−1_(38.23,21.08,4.086), *C*_0_(50,19.28,4.683), *C*_1_(61.77,21.08,4.086), *C*_2_(77.9,26.63,2.234), *C*_3_(100,29.65,1.228). The figure of this set of seven clouds is given in Fig 3. The direct impact matrix between criteria the decision-maker gives is transformed into the corresponding cloud matrices, and the results are shown in Table 6.

**Table 6:** The direct influence matrix processed using the cloud model.

$C_{i}$	First Block (C1-C6)						Second Block (C7-C12)					
	$C_{1}$	$C_{2}$	$C_{3}$	$C_{4}$	$C_{5}$	$C_{6}$	$C_{7}$	$C_{8}$	$C_{9}$	$C_{10}$	$C_{11}$	$C_{12}$
*C*_1_	(0,29.65,1.228)	(100,29.65,1.228)	(100,29.65,1.228)	(22.1,26.63,2.234)	(77.9,26.63,2.234)	(77.9,26.63,2.234)	(22.1,26.63,2.234)	(77.9,26.63,2.234)	(100,29.65,1.228)	(22.1,26.63,2.234)	(61.77,21.08,4.086)	(77.9,26.63,2.234)
*C*_2_	(100,29.65,1.228)	(0,29.65,1.228)	(50,19.28,4.683)	(100,29.65,1.228)	(100,29.65,1.228)	(100,29.65,1.228)	(0,29.65,1.228)	(77.9,26.63,2.234)	(50,19.28,4.683)	(50,19.28,4.683)	(100,29.65,1.228)	(100,29.65,1.228)
*C*_3_	(77.9,26.63,2.234)	(100,29.65,1.228)	(0,29.65,1.228)	(100,29.65,1.228)	(100,29.65,1.228)	(100,29.65,1.228)	(77.9,26.63,2.234)	(100,29.65,1.228)	(0,29.65,1.228)	(0,29.65,1.228)	(0,29.65,1.228)	(77.9,26.63,2.234)
*C*_4_	(100,29.65,1.228)	(77.9,26.63,2.234)	(100,29.65,1.228)	(0,29.65,1.228)	(100,29.65,1.228)	(100,29.65,1.228)	(50,19.28,4.683)	(100,29.65,1.228)	(100,29.65,1.228)	(77.9,26.63,2.234)	(50,19.28,4.683)	(100,29.65,1.228)
*C*_5_	(100,29.65,1.228)	(77.9,26.63,2.234)	(100,29.65,1.228)	(77.9,26.63,2.234)	(0,29.65,1.228)	(77.9,26.63,2.234)	(38.23,21.08,4.086)	(100,29.65,1.228)	(0,29.65,1.228)	(0,29.65,1.228)	(61.77,21.08,4.086)	(61.77,21.08,4.086)
*C*_6_	(0,29.65,1.228)	(0,29.65,1.228)	(61.77,21.08,4.086)	(100,29.65,1.228)	(77.9,26.63,2.234)	(0,29.65,1.228)	(22.1,26.63,2234)	(61.77,21.08,4.086)	(0,29.65,1.228)	(0,29.65,1.228)	(61.77,21.08,4.086)	(100,29.65,1.228)
*C*_7_	(0,29.65,1.228)	(22.1,26.63,2.234)	(50,19.28,4.683)	(77.9,26.63,2.234)	(0,29.65,1.228)	(77.9,26.63,2.234)	(0,29.65,1.228)	(0,29.65,1.228)	(77.9,26.63,2.234)	(22.1,26.63,2.234)	(0,29.65,1.228)	(77.9,26.63,2.234)
*C*_8_	(38.23,21.08,4.086)	(77.9,26.63,2.234)	(100,29.65,1.228)	(50,19.28,4.683)	(100,29.65,1.228)	(61.77,21.08,4.086)	(0,29.65,1.228)	(0,29.65,1.228)	(22.1,26.63,2.234)	(0,29.65,1.228)	(22.1,26.63,2.234)	(61.77,21.08,4.086)
*C*_9_	(22.1,26.63,2.234)	(50,19.28,4.683)	(0,29.65,1.228)	(100,29.65,1.228)	(77.9,26.63,2.234)	(100,29.65,1.228)	(22.1,26.63,2.234)	(22.1,26.63,2.234)	(0,29.65,1.228)	(22.1,26.63,2.234)	(22.1,26.63,2.234)	(100,29.65,1.228)
*C*_10_	(0,29.65,1.228)	(0,29.65,1.228)	(0,29.65,1.228)	(61.77,21.08,4.086)	(50,19.28,4.683)	(0,29.65,1.228)	(0,29.65,1.228)	(0,29.65,1.228)	(100,29.65,1.228)	(0,29.65,1.228)	(100,29.65,1.228)	(77.9,26.63,2.234)
*C*_11_	(38.23,21.08,4.086)	(77.9,26.63,2.234)	(50,19.28,4.683)	(77.9,26.63,2.234)	(0,29.65,1.228)	(77.9,26.63,2.234)	(0,29.65,1.228)	(77.9,26.63,2.234)	(100,29.65,1.228)	(50,19.28,4.683)	(0,29.65,1.228)	(100,29.65,1.228)
*C*_12_	(61.77,21.08,4.086)	(100,29.65,1.228)	(77.9,26.63,2.234)	(100,29.65,1.228)	(77.9,26.63,2.234)	(100,29.65,1.228)	(50,19.28,4.683)	(77.9,26.63,2.234)	(100,29.65,1.228)	(77.9,26.63,2.234)	(100,29.65,1.228)	(0,29.65,1.228)

**Fig 4:**
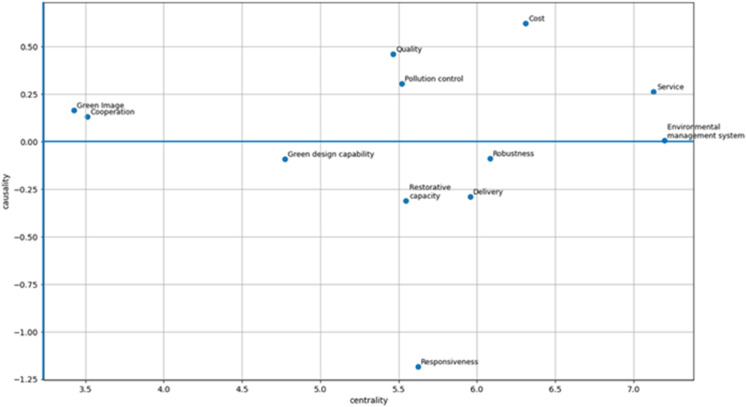
The causal diagram of supplier evaluation criteria.

The weights of the criteria are calculated according to Eqs (16)–(20) as *w*_1_ = 0.082, *w*_2_ = 0.094, *w*_3_ = 0.089, *w*_4_ = 0.106, *w*_5_ = 0.091, *w*_6_ = 0.086, *w*_7_ = 0.058, *w*_8_ = 0.083, *w*_9_ = 0.071, *w*_10_ = 0.051, *w*_11_ = 0.082, *w*_12_ = 0.10. Fig 4 depicts the causal diagram of sub-criteria. The results show that quality, cost, service, etc., have effects on delivery, robustness, responsiveness, etc.

**Phase 2: Prioritizing suppliers using the C-DEMATEL-TODIM approach** First, obtain the decision-makers’ linguistic multi-attribute decision matrix for suppliers *A*_1_-*A*_4_ (Table 3) and get its transformed cloud matrix as in Phase 2, as shown in Table 7. The reference weight is $w^{*}=\max\{w_{j}|1\leq j\leq 12\}=0.107$. According to Eq (21), the relative weight is $w^{\prime }=(0.766,0.879,0.832,0.991,0.850,0.804,0.542,0.776,0.664,0.477,0.766,1.000)$.

**Table 7:** Linguistic multi-attribute decision matrix processed using the cloud model.

	$C_{1}$	$C_{2}$	$C_{3}$	$C_{4}$	$C_{5}$	$C_{6}$	$C_{7}$	$C_{8}$	$C_{9}$	$C_{10}$	$C_{11}$	$C_{12}$
*A*_1_	(100,29.65,1.228)	(0,29.65,1.228)	(100,29.65,1.228)	(77.9,26.63,2.234)	(50,19.28,4.683)	(77.9,26.63,2.234)	(50,19.28,4.683)	(0,29.65,1.228)	(50,19.28,4.683)	(77.9,26.63,2.234)	(100,29.65,1.228)	(22.10,26.63,2.234)
*A*_2_	(100,29.65,1.228)	(61.77,21.08,4.086)	(77.9,26.63,2.234)	(61.77,21.08,4.086)	(77.9,26.63,2.234)	(77.9,26.63,2.234)	(0,29.65,1.228)	(77.9,26.63,2.234)	(22.10,26.63,2.234)	(50,19.28,4.683)	(100,29.65,1.228)	(100,29.65,1.228)
*A*_3_	(100,29.65,1.228)	(77.9,26.63,2.234)	(38.23,21.08,4.086)	(100,29.65,1.228)	(38.23,21.08,4.086)	(100,29.65,1.228)	(22.10,26.63,2.234)	(77.9,26.63,2.234)	(38.23,21.08,4.086)	(77.9,26.63,2.234)	(100,29.65,1.228)	(77.9,26.63,2.234)
*A*_4_	(77.9,26.63,2.234)	(100,29.65,1.228)	(0,29.65,1.228)	(38.23,21.08,4.086)	(77.9,26.63,2.234)	(100,29.65,1.228)	(100,29.65,1.228)	(77.9,26.63,2.234)	(38.23,21.08,4.086)	(77.9,26.63,2.234)	(77.9,26.63,2.234)	(0,29.65,1.228)

Taking the loss decay coefficient θ=1. According to Eq (22), calculate the dominance degree $\phi_{j}\left(A_{i},A_{l}\right)\;(i,l=1,2,3,4)$ of alternative *A*_*i*_ relative to alternative *A*_*l*_ under the criteria *C*_*j*_. For instance, based on criterion *C*_1_, alternative *A*_1_’s dominance over alternative *A*_4_ is $\phi_{1}\left(A_{1},A_{4}\right)=0.010$. In a similar manner, Table 8 displays the matrix of dominance degree under each criterion. Calculate the dominance degree $\delta_{j}\left(A_{i},A_{l}\right)\;(i,l=1,2,3,4)$ of alternative *A*_*i*_ over alternative *A*_*l*_ according to Eq (24), as shown in Table 9. Finally, according to Eq (25), the overall dominance degree of each alternative is calculated as $T\left(A_{1}\right)=0$, $T\left(A_{2}\right)=0.514$, $T\left(A_{3}\right)=1$, $T\left(A_{4}\right)=0.508$. According to the overall dominance degree of each alternative, the alternatives are ranked as: $A_{3}>A_{2}>A_{4}>A_{1}$.

**Table 8:** Dominance matrix regarding each criterion.

$A_{i}$	$C_{1}$				$C_{2}$				…	$C_{11}$				$C_{12}$			
	$A_{1}$	$A_{2}$	$A_{3}$	$A_{4}$	$A_{1}$	$A_{2}$	$A_{3}$	$A_{4}$		$A_{1}$	$A_{2}$	$A_{3}$	$A_{4}$	$A_{1}$	$A_{2}$	$A_{3}$	$A_{4}$
*A*_1_	0.000	0.000	0.000	0.010	0.000	–2.645	–2.806	–3.262	…	0.000	0.000	0.000	0.010	0.000	–1.992	–1.706	0.080
*A*_2_	0.000	0.000	0.000	0.010	0.249	0.000	–0.455	–0.403	…	0.000	0.000	0.000	0.010	0.213	0.000	0.012	0.327
*A*_3_	0.000	0.000	0.000	0.010	0.264	0.043	0.000	–0.115	…	0.000	0.000	0.000	0.010	0.183	–0.108	0.000	0.281
*A*_4_	–0.123	–0.123	–0.123	0.000	0.307	0.038	0.011	0.000	…	–0.123	–0.123	–0.123	0.000	–0.746	–3.057	–2.630	0.000

**Table 9:** Relative dominance matrix of each alternative.

	$A_{1}$	$A_{2}$	$A_{3}$	$A_{4}$
*A*_1_	0.000	–7.552	–7.448	-6.478
*A*_2_	–4.912	0.000	–3.365	–5.692
*A*_3_	–3.254	–0.835	0.000	–2.795
*A*_4_	–4.888	–4.261	–4.916	0.000

### Effect of attenuation factor of the losses

**Fig 5:**
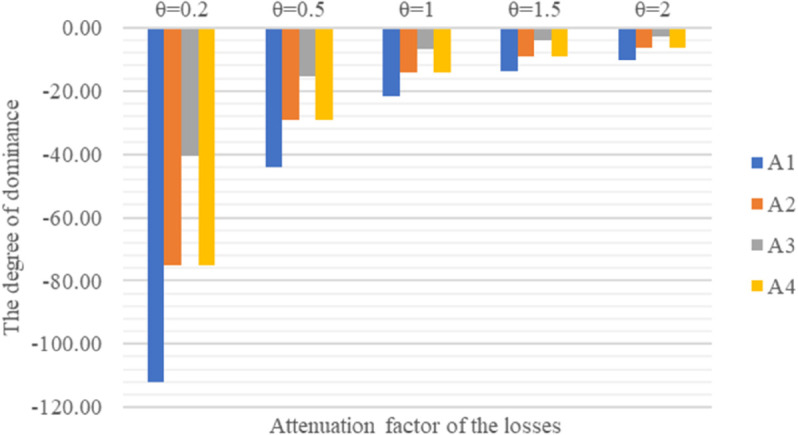
Sensitivity analysis plot.

Prospect theory suggests that individuals are more sensitive to losses than gains, and it is recommended to take θ<1. However, in most applications of the TODIM method, 1≤θ<2.5 is commonly selected. This is because the coefficient *θ* significantly influences the ranking of the programs. By choosing a larger *θ*, programs with a higher overall dominance degree can yield a greater gain, even if they have losses on certain programs. According to the case of this paper, by selecting different attenuation factors of the losses *θ*, and calculating the overall dominance degree and ranking results of each alternative, as shown in Fig 5.

According to Table 10, with the change of attenuation factor *θ*, the ranking of alternatives *A*_2_ and *A*_4_ changes accordingly. At θ=0.2 and θ=2.0, the dominance degree of *A*_2_ and *A*_4_ respectively relative to other programs under all criteria is shown in Table 11.

**Table 10:** Overall dominance and ranking orders of alternatives with different values of θ.

$A_{i}$	θ=0.2		θ=0.5		θ=1		θ=1.5		θ=2	
	$T(A_{i})$	Rank	*T*(*A*_*i*_)	Rank	$T(A_{i})$	Rank	$T(A_{i})$	Rank	$T(A_{i})$	Rank
*A*_1_	0	4	0	4	0	4	0	4	0	4
*A*_2_	0.514	3	0.514	3	0.514	2	0.514	2	0.514	2
*A*_3_	1	1	1	1	1	1	1	1	1	1
*A*_4_	0.515	2	0.516	2	0.508	3	0.504	3	0.501	3

**Table 11:** Dominance degree of *A*_2_ and *A*_4_ over others regarding each criterion with θ = 0.2 and θ = 2.

$C_{i}$	$A_{2}$						$A_{4}$					
	θ=0.2			θ=2			θ=0.2			θ=2		
	$A_{1}$	$A_{3}$	$A_{4}$	$A_{1}$	$A_{3}$	$A_{4}$	$A_{1}$	$A_{2}$	$A_{3}$	$A_{1}$	$A_{2}$	$A_{3}$
*C*_1_	0.000	0.000	0.010	0.000	0.000	0.010	–0.616	–0.616	–0.616	–0.062	–0.062	–0.062
*C*_2_	0.249	–2.275	–2.015	0.249	–0.227	–0.202	0.307	0.038	0.011	0.307	0.038	0.011
*C*_3_	–0.591	0.044	0.055	–0.059	0.044	0.055	–16.760	–3.108	–8.411	–1.676	–0.311	–0.841
*C*_4_	–2.142	–1.898	0.077	–0.214	–0.190	0.077	–2.200	–3.600	–2.800	–0.225	–0.362	–0.285
*C*_5_	0.027	0.044	0.000	0.027	0.044	0.000	0.027	0.000	0.044	0.027	0.000	0.044
*C*_6_	0.000	–0.602	–0.602	0.000	–0.060	–0.060	0.010	0.010	0.000	0.010	0.010	0.000
*C*_7_	–14.348	–5.067	–20.761	–1.435	–0.507	–2.076	0.018	0.241	0.157	0.018	0.241	0.157
*C*_8_	0.248	0.000	0.000	0.248	0.000	0.000	0.248	0.000	0.000	0.248	0.000	0.000
*C*_9_	–9.150	–5.465	–5.465	–0.915	–0.546	–0.546	–3.514	0.078	0.000	–0.351	0.078	0.000
*C*_10_	–2.015	–2.015	–2.015	–0.202	–0.202	–0.202	0.000	0.021	0.000	0.000	0.021	0.000
*C*_11_	0.000	0.000	0.010	0.000	0.000	0.0101	–0.616	–0.616	–0.616	–0.062	–0.062	–0.062
*C*_12_	0.213	0.012	0.327	0.213	0.012	0.327	–3.731	–15.285	–13.150	–0.373	–1.529	–1.315

From Eq (12) and Table 11, the parameter *θ* controls the effect of losses, and the gains of this alternative relative to the others are unaffected by the change in *θ*. *A*_4_ has losses in the less weighted criteria *C*_7_ and *C*_10_, and a large degree of superiority in the more weighted criteria *C*_4_ and *C*_12_, both of which have a large degree of superiority. When θ=2.0, the impact of these losses is reduced, so that the gains of *A*_2_ are more important than the losses and greater gains can be achieved; the overall dominance degree of program *A*_4_ is larger than *A*_2_ at θ=2.0; when θ=0.2, as can be seen from Table 10, these losses of *A*_4_ are seriously amplified, so that program *A*_2_ has a smaller gain relative to *A*_4_, but at the same time the losses are relatively small; the overall dominance degree of program *A*_2_ is larger than *A*_4_ at θ=0.2.

### Comparison analysis

The improved C-TODIM method proposed in this paper is compared with the cloud-TOPSIS method to confirm the validity and superiority of the proposed method. The weighted cloud decision matrix is computed using the cloud-TOPSIS method, which primarily employs the cloud distance measure algorithm. Each weighted cloud is compared with the positive and negative clouds, and the distance between each weighted cloud and the ideal cloud models is computed. This paper states that the TOPSIS method is used to calculate the weighted cloud decision matrix, calculated index weights, and relative closeness of each supplier. The relative closeness Ui of every alternative was calculated using this method. Table 12 displays the results.

**Table 12:** Close degree in cloud-TOPSIS of all candidate suppliers.

	$A_{1}$	$A_{2}$	$A_{3}$	$A_{4}$
$d_{i}^{+}$	0.094	0.034	0.023	0.097
$d_{i}^{-}$	0.071	0.118	0.116	0.080
*U*_*i*_	0.430	0.774	0.833	0.452

According to Table 11, the TOPSIS method ranks the suppliers as $A_{3}>A_{2}>A_{4}>A_{1}$. Cloud-TOPSIS ranks the four suppliers in the same order as the C-TODIM method used in this paper. This result indicates that the proposed methodology is fully correlated with the other established methods, which validates the results obtained in the case study.

### Evaluation

In order to assess the results of the case studies and the proposed method, we first vary the loss recession factor to perform a sensitivity analysis. This is mainly to confirm that the variation of the method *θ* is robust. Table 9 shows the results for *θ*, taking five different values. From Table 9, it is easy to realize that the ranking results of alternative suppliers may vary with the value of *θ*. The ranking results when θ<1 are the same, $A_{3}>A_{4}>A_{2}>A_{1}$; when θ ≥ 1, the ranking results change, $A_{3}>A_{2}>A_{4}>A_{1}$. In all five cases, since *θ* is the key coefficient reflecting the decision-maker’s loss preference, the change in supplier ranking is highly dependent on the value of *θ*. The most typical reason for this variation is described as follows. When θ<1, the loss is amplified, and the amplification increases as *θ* decreases. When θ ≥ 1, the loss is attenuated, and the degree of attenuation increases with *θ*. The attenuation of the loss results in a better ranking of *A*_2_ than *A*_4_. This approach can lead to a more reasonable ranking based on the decision-maker’s preference for loss.

Given that the proposed approach depends on a comparatively recent MCDM method, we additionally conducted a comparative analysis to assess the stability of the outcomes. In this context, comparative analysis means assessing whether decisions are affected when the decision-making process changes, such as when a different MCDM method is used. We compare our method with Cloud-TOPSIS: Cloud-TOPSIS was chosen for comparison because it is easy to use and widely available and because it is representative of recently proposed MCDM methods and is heavily cited in the literature.

From the computational results, it can be seen that when θ ≥ 1, the sorting order of the Cloud-TOPSIS method is the same as that of this paper’s method, while when θ<1, it is different from that of this paper’s method. The same sorting order indicates that the proposed method is effective and reasonable, and the different sorting results indicate the superiority of the method proposed in this paper.

These results can be interpreted in this way. The method suggested in this paper stands out because it modifies the value of the parameter *θ* to account for the subject’s psychological behavior. The degree of damage avoidance of the decision-maker decreases with the increase of the value of *θ*. That is, when the value of parameter *θ* reaches 1, the psychological behavior of the decision-maker can be ignored. In this case, we do not consider the psychological behavior of the decision-maker, and thus, the method proposed in this paper can produce the same sorting order as the existing methods in the medium because they do not consider the psychological behavior of the decision-maker. Obviously, this can show the effectiveness of the method proposed in this paper. When making decisions, decision-makers are always subject to limitations in their rationality. Therefore, it is more appropriate to focus on the psychological behavior of decision makers in this paper.

In conclusion, compared with the Cloud-TOPSIS method, the developed method can also consider the psychological behavior of the decision-maker by reducing the parameter *θ* to a certain value and weakening the psychological behavior of the decision-maker by increasing the parameter *θ* to a certain value.

### Practical and theoretical implications

Based on the discussion above, this study offers significant practical implications for both managers and policymakers in the field of sustainable supply chain management. By integrating cloud model theory with the C-DEMATEL-TODIM framework, the research provides a structured and adaptive decision-support tool that effectively handles the ambiguity, randomness, and psychological factors inherent in green-resilient supplier selection. This approach enables companies to more accurately evaluate suppliers based on both environmental and resilience criteria, supporting strategic alignment with UN SDGs while enhancing operational continuity under disruptions. Moreover, the proposed artifact emphasizes mutual value creation across stakeholders by incorporating influential relationships among criteria and accounting for bounded rationality in decision-making. Enterprises can leverage this methodology not only to improve their supplier selection processes but also to foster transitions toward more sustainable business models. By offering a scalable and practical analytical tool, this study assists firms in mitigating risks associated with product misuse, resource instability, and regulatory changes, thereby contributing to broader sustainability initiatives and stakeholder-based care principles in real-world settings.

This study also contributes to the advancement of MCDM methodologies, particularly through the novel integration of cloud model with DEMATEL and TODIM, offering a new approach to handling qualitative and uncertain information in sustainable supply chain contexts. By introducing the C-DEMATEL-TODIM framework, this research provides a theoretical mechanism that simultaneously captures the fuzziness and randomness of linguistic evaluations, models influential relationships among criteria, and incorporates psychological preferences of decision-makers—addressing key limitations in existing green and resilient supplier selection literature. The proposed hybrid model extends the application of cloud theory and behavioral MCDM methods in operational management contexts, demonstrating how computational tools can better mirror real-world cognitive and environmental complexities.

Furthermore, this study offers theoretical insights into the integration of corporate sustainability and stakeholder theory within supplier evaluation processes. By embedding care-based and mutual value-driven principles into a quantifiable decision structure, the framework supports the transition toward sustainable business models and enhances the understanding of how firms can operationalize sustainability goals in supplier management. The methodology developed here is adaptable across industries where sustainability and resilience are critical, providing a replicable foundation for future research aimed at balancing environmental, social, and economic criteria under uncertainty. These contributions respond to growing scholarly calls for more psychologically realistic and computationally robust models in sustainable operations management and offer a generalized theoretical artifact that bridges theoretical rigor with practical applicability.

## Conclusion

While the evaluation and selection of sustainable suppliers are critical problems, existing research often fails to integrate green and resilience criteria systematically and seldom addresses the complex interrelationships among these criteria. Moreover, conventional MCDM methods are limited in handling the inherent fuzziness of linguistic evaluations and frequently overlook DMs’ psychological preferences and risk perceptions. To bridge these gaps, this study proposes a novel fuzzy MCDM approach that combines cloud model with the DEMATEL and TODIM techniques for the comprehensive evaluation of green-resilient suppliers. First, a comprehensive literature review and expert consultations were conducted to identify and classify evaluation criteria into a structured system comprising three main dimensions—general, resilience, and green—which are further broken down into 12 sub-criteria. Subsequently, the C-DEMATEL method was applied to model the influential relationships among criteria. Linguistic evaluations from experts were converted into a direct-influence matrix via cloud model, and criterion weights were derived accordingly. Within this phase, “Robustness” (under the resilience dimension) and “Environmental Management System” (under the green dimension) emerged as the most influential sub-criteria. In the third phase, the C-TODIM method was employed to rank potential suppliers under fuzzy environments, incorporating DMs’ risk attitudes through a psychological attenuation factor. Finally, a sensitivity analysis on the risk coefficient *θ* in TODIM and a comparative study with the Cloud-TOPSIS method were carried out to validate the stability and reliability of the proposed framework.

From a managerial perspective, this paper emphasizes the importance of green-resilient in supplier selection, which is verified through case studies. By applying the methodology proposed in this paper, enterprises can prioritize suppliers with green design and production capabilities, strong environmental pollution control capabilities, and sound environmental management systems to promote the greening and sustainable development of the supply chain. Concurrently, enterprises may also prioritize the resilience-building of their supply chains, collectively addressing external risks and challenges through enhanced collaboration and communication with suppliers. By implementing a scientific and comprehensive supplier selection and management process, enterprises can establish a more stable, efficient, and environmentally conscious supply chain system, thereby enhancing the quality and competitiveness of their products. This approach will assist enterprises in distinguishing themselves in the competitive market and attaining a greater market share and consumer trust. Concurrently, the enterprise’s green and resilient image will be enhanced, leading to greater social recognition and support for the enterprise.

However, this study still has several limitations. Future research may explore more advanced mathematical tools and models, such as machine learning and deep learning techniques, to further improve the assessment’s accuracy and efficiency. Additionally, interdisciplinary integration will become a prominent trend, with the incorporation of theories and methods from fields such as economics, environmental science, and sociology to provide more comprehensive support for the model. Furthermore, environmental footprint assessment and resource efficiency analysis can be integrated to quantify the green-resilient performance of suppliers. Complex network theory and risk propagation models can be employed to analyze the resilience level of supply chains. Concurrently, research dynamics in emerging fields such as sustainable technology innovation and green supply chain management can be concentrated on to provide novel concepts and solutions for green and resilient supplier selection.

## Supporting information

S1 Dataset Data used in this Case study. (DOCX)
