# Supplementary material for: A cloud-based multi-criteria decision-making framework for green-resilient supplier selection of manufacturing industry
Source: PLoS One. 2026 Feb 19;21(2):e0343165. doi: 10.1371/journal.pone.0343165 (PMC12919935; doi:10.1371/journal.pone.0343165)
Supplement: S1 Dataset — (DOCX) [file pone.0343165.s001.docx]

The raw data for this manuscript is presented in the table below.

Table 1 A review of the related literature

| Researchers | Methodology | Advantage | | | | |
| --- | --- | --- | --- | --- | --- | --- |
|  |  | Randomness | Ambiguity | Interaction between criteria | The psychological behavior of decision-makers | Reduce the computational difficulty |
| ([Mohammed, 2020](#_ENREF_30)) | DEMATEL- VIKOR |  |  | √ |  |  |
| ([Fallahpour et al., 2021](#_ENREF_15)) | FDEMATEL+FBWM+FANP+FIS |  | √ | √ |  |  |
| ([Hailiang et al., 2023](#_ENREF_19)) | FAHP+DEA+TOPSIS | √ | √ |  | √ | √ |
| ([Sen et al., 2017](#_ENREF_45)) | fuzzy set theory+simplified version of TODIM and PROMETHEE | √ | √ |  | √ | √ |
| ([Afrasiabi et al., 2022](#_ENREF_2)) | fuzzy best-worst method (FBWM)+fuzzy grey relational analysis (GRA)-TOPSIS | √ | √ |  |  |  |
| ([Abedian et al., 2023](#_ENREF_1)) | fuzzy set theory + data envelopment analysis (DEA) |  | √ |  |  | √ |
| this study | C-DEMATEL-TODIM | √ | √ | √ | √ | √ |

Table 3. The linguistic multi-attribute decision matrix given by the expert group

|  | C_1_ | C_2_ | C_3_ | C_4_ | C_5_ | C_6_ | C_7_ | C_8_ | C_9_ | C_10_ | C_11_ | C_12_ |
| --- | --- | --- | --- | --- | --- | --- | --- | --- | --- | --- | --- | --- |
| A_1_ | VG | VP | VG | G | F | G | F | VP | M | G | VG | P |
| A _2_ | VG | MG | G | MG | G | G | VP | G | P | F | VG | VG |
| A _3_ | VG | G | MP | VG | MP | VG | P | G | MP | G | VG | G |
| A _4_ | G | VG | VP | MP | G | VG | VG | G | MP | G | G | VP |

Table 4. Direct impact matrix between criteria given by the expert group

|  | C_1_ | C_2_ | C_3_ | C_4_ | C_5_ | C_6_ | C_7_ | C_8_ | C_9_ | C_10_ | C_11_ | C_12_ |
| --- | --- | --- | --- | --- | --- | --- | --- | --- | --- | --- | --- | --- |
| C_1_ | VL | VH | VH | L | H | H | L | H | VH | L | MH | H |
| C_2_ | VH | VL | M | VH | VH | VH | VL | H | M | M | VH | VH |
| C_3_ | H | VH | VL | VH | VH | VH | H | VH | VL | VL | VL | H |
| C_4_ | VH | H | VH | VL | VH | VH | M | VH | VH | H | M | VH |
| C_5_ | VH | H | VH | H | VL | H | ML | VH | VL | VL | MH | MH |
| C_6_ | VL | VL | MH | VH | H | VL | L | MH | VL | VL | MH | VH |
| C­_7_ | VL | L | M | H | VL | H | VL | VL | H | L | VL | H |
| C_8_ | ML | H | VH | M | VH | MH | VL | VL | L | VL | L | MH |
| C­_9_ | L | M | VL | VH | H | VH | L | L | VL | L | L | VH |
| C_10_ | VL | VL | VL | MH | M | VL | VL | VL | VH | VL | VH | H |
| C_11_ | ML | H | M | H | VL | H | VL | H | VH | M | VL | VH |
| C_12_ | MH | VH | H | VH | H | VH | M | H | VH | H | VH | VL |

|  | C_1_ | C_2_ | C_3_ | C_4_ | C_5_ | C_6_ |
| --- | --- | --- | --- | --- | --- | --- |
| C_1_ | (0,29.65,1.228) | (100,29.65,1.228) | (100,29.65,1.228) | (22.1,26.63,2.234) | (77.9,26.63,2.234) | (77.9,26.63,2.234) |
| C_2_ | (100,29.65,1.228) | (0,29.65,1.228) | (50,19.28,4.683) | (100,29.65,1.228) | (100,29.65,1.228) | (100,29.65,1.228) |
| C_3_ | (77.9,26.63,2.234) | (100,29.65,1.228) | (0,29.65,1.228) | (100,29.65,1.228) | (100,29.65,1.228) | (100,29.65,1.228) |
| C_4_ | (100,29.65,1.228) | (77.9,26.63,2.234) | (100,29.65,1.228) | (0,29.65,1.228) | (100,29.65,1.228) | (100,29.65,1.228) |
| C_5_ | (100,29.65,1.228) | (77.9,26.63,2.234) | (100,29.65,1.228) | (77.9,26.63,2.234) | (0,29.65,1.228) | (77.9,26.63,2.234) |
| C_6_ | (0,29.65,1.228) | (0,29.65,1.228) | (61.77,21.08,4.086) | (100,29.65,1.228) | (77.9,26.63,2.234) | (0,29.65,1.228) |
| C­_7_ | (0,29.65,1.228) | (22.1,26.63,2.234) | (50,19.28,4.683) | (77.9,26.63,2.234) | (0,29.65,1.228) | (77.9,26.63,2.234) |
| C_8_ | (38.23,21.08,4.086) | (77.9,26.63,2.234) | (100,29.65,1.228) | (50,19.28,4.683) | (100,29.65,1.228) | (61.77,21.08,4.086) |
| C­_9_ | (22.1,26.63,2.234) | (50,19.28,4.683) | (0,29.65,1.228) | (100,29.65,1.228) | (77.9,26.63,2.234) | (100,29.65,1.228) |
| C_10_ | (0,29.65,1.228) | (0,29.65,1.228) | (0,29.65,1.228) | (61.77,21.08,4.086) | (50,19.28,4.683) | (0,29.65,1.228) |
| C_11_ | (38.23,21.08,4.086) | (77.9,26.63,2.234) | (50,19.28,4.683) | (77.9,26.63,2.234) | (0,29.65,1.228) | (77.9,26.63,2.234) |
| C_12_ | (61.77,21.08,4.086) | (100,29.65,1.228) | (77.9,26.63,2.234) | (100,29.65,1.228) | (77.9,26.63,2.234) | (100,29.65,1.228) |
|  | C_7_ | C_8_ | C_9_ | C_10_ | C_11_ | C_12_ |
| C_1_ | (22.1,26.63,2.234) | (77.9,26.63,2.234) | (100,29.65,1.228) | (22.1,26.63,2.234) | (61.77,21.08,4.086) | (77.9,26.63,2.234) |
| C_2_ | (0,29.65,1.228) | (77.9,26.63,2.234) | (50,19.28,4.683) | (50,19.28,4.683) | (100,29.65,1.228) | (100,29.65,1.228) |
| C_3_ | (77.9,26.63,2.234) | (100,29.65,1.228) | (0,29.65,1.228) | (0,29.65,1.228) | (0,29.65,1.228) | (77.9,26.63,2.234) |
| C_4_ | (50,19.28,4.683) | (100,29.65,1.228) | (100,29.65,1.228) | (77.9,26.63,2.234) | (50,19.28,4.683) | (100,29.65,1.228) |
| C_5_ | (38.23,21.08,4.086) | (100,29.65,1.228) | (0,29.65,1.228) | (0,29.65,1.228) | (61.77,21.08,4.086) | (61.77,21.08,4.086) |
| C_6_ | (22.1,26.63,2234) | (61.77,21.08,4.086) | (0,29.65,1.228) | (0,29.65,1.228) | (61.77,21.08,4.086) | (100,29.65,1.228) |
| C­_7_ | (0,29.65,1.228) | (0,29.65,1.228) | (77.9,26.63,2.234) | (22.1,26.63,2.234) | (0,29.65,1.228) | (77.9,26.63,2.234) |
| C_8_ | (0,29.65,1.228) | (0,29.65,1.228) | (22.1,26.63,2.234) | (0,29.65,1.228) | (22.1,26.63,2.234) | (61.77,21.08,4.086) |
| C­_9_ | (22.1,26.63,2.234) | (22.1,26.63,2.234) | (0,29.65,1.228) | (22.1,26.63,2.234) | (22.1,26.63,2.234) | (100,29.65,1.228) |
| C_10_ | (0,29.65,1.228) | (0,29.65,1.228) | (100,29.65,1.228) | (0,29.65,1.228) | (100,29.65,1.228) | (77.9,26.63,2.234) |
| C_11_ | (0,29.65,1.228) | (77.9,26.63,2.234) | (100,29.65,1.228) | (50,19.28,4.683) | (0,29.65,1.228) | (100,29.65,1.228) |
| C_12_ | (50,19.28,4.683) | (77.9,26.63,2.234) | (100,29.65,1.228) | (77.9,26.63,2.234) | (100,29.65,1.228) | (0,29.65,1.228) |

Table 5 Cloud matrix after direct influence matrix transformation between crit

Table 6. Linguistic multi-attribute decision matrix transformed cloud matrix

|  | C_1_ | C_2_ | C_3_ | C_4_ | C_5_ | C_6_ | C_7_ | C_8_ | C_9_ | C_10_ | C_11_ | C_12_ |
| --- | --- | --- | --- | --- | --- | --- | --- | --- | --- | --- | --- | --- |
| A_1_ | (100,29.65,1.228) | (0,29.65,1.228) | (100,29.65,1.228) | (77.9,26.63,2.234) | (50,19.28,4.683) | (77.9,26.63,2.234) | (50,19.28,4.683) | (0,29.65,1.228) | (50,19.28,4.683) | (77.9,26.63,2.234) | (100,29.65,1.228) | (22.10,26.63,2.234) |
| A _2_ | (100,29.65,1.228) | (61.77,21.08,4.086) | (77.9,26.63,2.234) | (61.77,21.08,4.086) | (77.9,26.63,2.234) | (77.9,26.63,2.234) | (0,29.65,1.228) | (77.9,26.63,2.234) | (22.10,26.63,2.234) | (50,19.28,4.683) | (100,29.65,1.228) | (100,29.65,1.228) |
| A _3_ | (100,29.65,1.228) | (77.9,26.63,2.234) | (38.23,21.08,4.086) | (100,29.65,1.228) | (38.23,21.08,4.086) | (100,29.65,1.228) | (22.10,26.63,2.234) | (77.9,26.63,2.234) | (38.23,21.08,4.086) | (77.9,26.63,2.234) | (100,29.65,1.228) | (77.9,26.63,2.234) |
| A _4_ | (77.9,26.63,2.234) | (100,29.65,1.228) | (0,29.65,1.228) | (38.23,21.08,4.086) | (77.9,26.63,2.234) | (100,29.65,1.228) | (100,29.65,1.228) | (77.9,26.63,2.234) | (38.23,21.08,4.086) | (77.9,26.63,2.234) | (77.9,26.63,2.234) | (0,29.65,1.228) |

Table 7. Dominance matrix regarding each criterion

Table 8. Relative dominance matrix of each alternative

|  | A_1_ | A _2_ | A _3_ | A _4_ |
| --- | --- | --- | --- | --- |
| A_1_ | 0.000 | -7.552 | -7.448 | -6.478 |
| A _2_ | -4.912 | 0.000 | -3.365 | -5.692 |
| A _3_ | -3.254 | -0.835 | 0.000 | -2.795 |
| A _4_ | -4.888 | -4.261 | -4.916 | 0.000 |

Table 9. Overall dominance and ranking orders of alternatives with different values of θ

| A_i_ | θ=0.2 | | θ=0.5 | | θ=1 | | θ=1.5 | | θ=2 | |
| --- | --- | --- | --- | --- | --- | --- | --- | --- | --- | --- |
|  | T(A_i_) | Rank | T(A_i_) | Rank | T(A_i_) | Rank | T(A_i_) | Rank | T(A_i_) | Rank |
| A_1_ | 0 | 4 | 0 | 4 | 0 | 4 | 0 | 4 | 0 | 4 |
| A _2_ | 0.514 | 3 | 0.514 | 3 | 0.514 | 2 | 0.514 | 2 | 0.514 | 2 |
| A _3_ | 1 | 1 | 1 | 1 | 1 | 1 | 1 | 1 | 1 | 1 |
| A _4_ | 0.515 | 2 | 0.516 | 2 | 0.508 | 3 | 0.504 | 3 | 0.501 | 3 |

Table 10. Dominance degree of A_2_ and A_4_ over others regarding each criterion with θ=0.2 and θ=2

| C_i_ | A _2_ | | | | | | A _4_ | | | | | |
| --- | --- | --- | --- | --- | --- | --- | --- | --- | --- | --- | --- | --- |
|  | θ=0.2 | | | θ=2 | | | θ=0.2 | | | θ=2 | | |
|  | A_1_ | A _3_ | A _4_ | A_1_ | A _3_ | A _4_ | A_1_ | A _2_ | A _3_ | A_1_ | A _2_ | A _3_ |
| C_1_ | 0.000 | 0.000 | 0.010 | 0.000 | 0.000 | 0.010 | -0.616 | -0.616 | -0.616 | -0.062 | -0.062 | -0.062 |
| C_2_ | 0.249 | -2.275 | -2.015 | 0.249 | -0.227 | -0.202 | 0.307 | 0.038 | 0.011 | 0.307 | 0.038 | 0.011 |
| C_3_ | -0.591 | 0.044 | 0.055 | -0.059 | 0.044 | 0.055 | -16.760 | -3.108 | -8.411 | -1.676 | -0.311 | -0.841 |
| C_4_ | -2.142 | -1.898 | 0.077 | -0.214 | -0.190 | 0.077 | -2.200 | -3.600 | -2.800 | -0.225 | -0.362 | -0.285 |
| C_5_ | 0.027 | 0.044 | 0.000 | 0.027 | 0.044 | 0.000 | 0.027 | 0.000 | 0.044 | 0.027 | 0.000 | 0.044 |
| C_6_ | 0.000 | -0.602 | -0.602 | 0.000 | -0.060 | -0.060 | 0.010 | 0.010 | 0.000 | 0.010 | 0.010 | 0.000 |
| C­_7_ | -14.348 | -5.067 | -20.761 | -1.435 | -0.507 | -2.076 | 0.018 | 0.241 | 0.157 | 0.018 | 0.241 | 0.157 |
| C_8_ | 0.248 | 0.000 | 0.000 | 0.248 | 0.000 | 0.000 | 0.248 | 0.000 | 0.000 | 0.248 | 0.000 | 0.000 |
| C­_9_ | -9.150 | -5.465 | -5.465 | -0.915 | -0.546 | -0.546 | -3.514 | 0.078 | 0.000 | -0.351 | 0.078 | 0.000 |
| C_10_ | -2.015 | -2.015 | -2.015 | -0.202 | -0.202 | -0.202 | 0.000 | 0.021 | 0.000 | 0.000 | 0.021 | 0.000 |
| C_11_ | 0.000 | 0.000 | 0.010 | 0.000 | 0.000 | 0.0101 | -0.616 | -0.616 | -0.616 | -0.062 | -0.062 | -0.062 |
| C_12_ | 0.213 | 0.012 | 0.327 | 0.213 | 0.012 | 0.327 | -3.731 | -15.285 | -13.150 | -0.373 | -1.529 | -1.315 |

Table 11 Close degree in cloud-TOPSIS of all candidate suppliers

|  | A_1_ | A _2_ | A _3_ | A _4_ |
| --- | --- | --- | --- | --- |
| $d_{i}^{+}$ | 0.094 | 0.034 | 0.023 | 0.097 |
| $d_{i}^{-}$ | 0.071 | 0.118 | 0.116 | 0.080 |
| $U_{i}$ | 0.430 | 0.774 | 0.833 | 0.452 |
